# Impact of residual feed intake on jejunal tight junction morphology and gene expression in slow-growing Korat chickens

**DOI:** 10.1016/j.psj.2025.105667

**Published:** 2025-08-08

**Authors:** Sukritta Kongthungmon, Saknarin Pengsanthia, Boonyarit Kamkrathok, Pramin Kaewsatuan, Wittawat Molee, Amonrat Molee

**Affiliations:** School of Animal Technology and Innovation, Institute of Agricultural Technology, Suranaree University of Technology, Nakhon Ratchasima, 30000, Thailand

**Keywords:** Slow-growing chicken, Residual feed intake, Feed efficiency, Tight junction, Jejunum

## Abstract

Improving feed efficiency (FE) in Korat chickens (KRC), a Thai slow-growing breed, is essential for reducing production costs. This study aimed to investigate gene and protein expression related to oxidative phosphorylation and tight junction pathways, as well as jejunal tight junction morphology in KRC with divergent residual feed intake (RFI). A total of 115 male KRC was raised individually and fed in three phases (starter, grower, and finisher). At 10 wk, RFI was calculated and used to select low-RFI (LRFI) and high-RFI (HRFI) groups (*n* = 15/group). Gene and protein expression were analyzed by real-time quantitative PCR and Western blot, respectively. Tight junction morphology was examined by Transmission Electron Microscope technique. LRFI chickens showed significantly lower RFI and FCR than HRFI chickens (*P* < 0.05) and exhibited upregulated expression of tight junction-related genes: Actinin Alpha 1 (ACTN1), Tubulin Alpha 3E (*P* < 0.05), while oxidative phosphorylation-related genes showed no significant differences. Additionally, the LRFI group demonstrated significantly higher ACTN1 protein expression (*P* < 0.05). Morphological analysis revealed that the LRFI group exhibited more compact and structurally intact tight junctions. These results indicate that improved intestinal barrier function and nutrient absorption in LRFI chickens may contribute to enhanced FE, highlighting their potential for genetic improvement in slow-growing chickens.

## Introduction

Improving feed efficiency (**FE**) is a critical goal in poultry production, particularly in slow-growing chicken breeds that are gaining popularity due to their meat quality and suitability for sustainable farming systems (Poompramun et al., 2021; Kaewsatuan et al., 2023). However, low FE in these breeds poses economic challenges for smallholder farmers and increases environmental burdens associated with feed use. Although slow-growing chicken breeds are generally favored for their superior meat texture and quality, they typically exhibit lower FE compared to fast-growing commercial lines, which poses a challenge for sustainable and cost-effective production (Fanatico et al., 2007; Mikulski et al., 2011). Understanding the biological mechanisms underlying FE variation in slow-growing chickens in this context could benefit broader efforts to optimize performance in indigenous and slow-growing poultry breeds. This study focused on Korat chickens (**KRC**), a representative slow-growing crossbreed developed in Thailand, as a model to investigate these mechanisms.

Feed conversion ratio (**FCR**) and residual feed intake (**RFI**) are critical parameters for evaluating FE. FCR, defined as the ratio of feed intake to body weight gain (**BWG**), often favors high-growth phenotypes despite increased feed consumption (Zamani et al., 2008; Willems et al., 2013). Conversely, RFI quantifies the deviation between actual and predicted FI, accounting for both metabolic maintenance and growth requirements (Aggrey et al., 2010; Xu et al., 2016). As an independent metric of growth rate, RFI enables precise genetic selection for feed-efficient phenotypes (Zamani et al., 2008; Willems et al., 2013; Xu et al., 2016). Therefore, RFI is a superior criterion for optimizing KRC breeding programs and other slow-growing chicken, enhancing sustainability, minimizing feed costs, and advancing the genetic basis of FE (Aggrey et al., 2010; Wen et al., 2018; Yi et al., 2018).

The digestive system is essential for converting feed into nutrients required for growth, maintenance, and reproduction, with the jejunum playing a key role in digestion, nutrient absorption, and immune regulation (Rodgers et al., 2012; Zhang et al., 2023). The gut barrier, composed of mucus, epithelial cells, tight junctions, and lamina propria, is vital for maintaining health and efficient nutrient assimilation. Feed particle size and digestion efficiency are also known to influence intestinal morphology and nutrient uptake, with finely ground diets improving digestive capacity and gut surface area in chickens (Rougière et al., 2009). Gene expression related to intestinal microvilli structure, particularly oxidative phosphorylation, and fat metabolism has been reported to be associated with FE in chickens (Yang et al., 2020; Xiao et al., 2021). In KRC, Sinpru et al. (2021) identified 56 FCR-associated genes involved in immune response, metabolism, and cellular development, indicating a multi-systemic influence on FE. Proteomic analysis has further supported this association by identifying 40 differentially abundant proteins related to glycolysis, peroxisome activity, and oxidative phosphorylation in chickens with divergent FE (Kaewsatuan et al., 2022). Among these, mitochondrial proteins such as *ATP synthase, H+ transporting, mitochondrial Fo complex subunit F6* (***ATP5J***) *ATP synthase, H+ transporting, mitochondrial Fo complex subunit D* (***ATP5H***) and *ATP synthase, H+ transporting, mitochondrial F1 complex, delta subunit* (***ATP5D***) are subunits of the ATP synthase (Complex V) that play essential roles in ATP synthesis through oxidative phosphorylation. These proteins are directly involved in the proton transport and catalytic conversion of ADP to ATP, which are fundamental for sustaining energy-requiring physiological processes, including nutrient transport, tissue growth, and cellular maintenance (Kong et al., 2016; Yang et al., 2020; Kaewsatuan et al., 2022). The efficiency of this energy conversion process is believed to reflect the animal’s capacity to utilize feed for productive purposes. Although the expression of these genes has been associated with FE (Kaewsatuan et al., 2022), it remains unclear whether such differences are evident in the jejunum, an intestinal segment critical for nutrient absorption. Understanding whether *ATP5J, ATP5H*, and *ATP5D* expression varies in the jejunal tissue of chickens with divergent FE would help clarify their role at the primary site of energy uptake. This knowledge gap limits their current application as precise molecular markers for FE in slow-growing chickens.

The tight junction pathway is essential for the physiological function of epithelial cells and influences nutrient absorption in the small intestine (Choct, 2009). The permeability of the intestinal barrier is regulated by the stability of tight junctions (Turner, 2009; Suzuki, 2013). Tight junction integrity and paracellular permeability have been linked to the regulation of the actin cytoskeleton and the strength of intercellular adhesion (Bruewer et al., 2004). Moreover, tight junction disruption under oxidative stress conditions has been shown to impair jejunal barrier function and growth performance in broilers (Chen et al., 2022). Furthermore, the expression of ACTN1, Solute Carrier Family 9 Member A3 regulator 1 (**SLC9A3R1**) and Tubulin Alpha 3E (**TUBA3E**) proteins has been associated with the tight junction pathway in KRC (Kaewsatuan et al., 2022). Based on these findings, we hypothesize that functional changes in oxidative phosphorylation and tight junction pathways, as reflected by genes and proteins in the jejunum, may contribute to FE in chickens.

To date, no studies have examined the impact of FE in slow-growing chickens on gene and protein expression related to oxidative phosphorylation, the tight junction pathway, and jejunal morphology. Thus, the objective of this study was to investigate gene and protein expression associated with the oxidative phosphorylation pathway, the tight junction pathway, and tight junction morphology in the jejunum of KRC with divergent FE. These findings provide valuable insights for designing selection programs aimed at enhancing the production efficiency of KRC while reducing feed costs and promoting environmental sustainability.

## Materials and methods

### Ethics statement

The animal protocols used in this study adhered to the guidelines approved by the Ethics Committee on Animal Use of Suranaree University of Technology (SUT), Nakhon Ratchasima, Thailand (Approval ID: SUT-IACUC-001/2021).

### Experimental animal and tissue collection

This study used 115 male KRC. The chickens were raised in individual cages. Feed and water were provided *ad libitum*. The commercial feed was divided into three types of experiment periods using a starter diet (21 % protein), a grower diet (19 % protein), and a finisher diet (17 % protein) at 0-3, 4-6, and 7-10 wk of age, respectively. FE data were calculated every week from body weight (BW) and FI. FCR was calculated as the ratio of FI to BWG. Metabolic body weight (MBW^0.75^) and BWG were determined based on BW measurements. RFI, was estimated using the equation: $RFI_{i}=FI_{i}-\left(\beta_{0}\;+\;\left(\beta_{1}MB{W^{0.75}}_{i}\right)+\left(\beta_{2}BWG_{i}\right)\right)$ where $FI_{i}\;$= actual feed intake of individual *i*, $MB{W^{0.75}}_{i}$ = metabolic body weight (body weight raised to the power of 0.75) of individual *i*, $BWG_{i}$ = body weight gain of individual *i*, β₀ is the regression intercept, β₁ and β₂ is the partial regression coefficient (Koch et al., 1963). At the age of 10 wk, RFI was analyzed and used to rank the chickens into 2 groups (*n* = 15/group): low-RFI (**LRFI**; −292.779 to −148.976 g) and high-RFI (**HRFI**; 165.490 to 388.364 g).

### Jejunal sample collection

At 10 wk of age, the LRFI and HRFI chickens were euthanized by electrical stunning and followed by exsanguination. After dissecting the jejunum, the submucosal layer was carefully separated from the underlying intestinal muscle using a sterile scalpel blade. The isolated submucosal tissue was collected into microtubes, snap-frozen in liquid nitrogen, and stored at −80°C for subsequent real-time PCR and Western blot techniques. In addition, the jejunal samples were cut into 1 cm segments and stored in 2.5 % glutaraldehyde at 4 °C for Transmission Electron Microscope (**TEM**; TECNAI 20, Philips, Ohio, USA) to investigate tight junction morphology.

### Total RNA extraction

The extraction of total RNA was performed as previously described (Sinpru et al., 2021). Briefly, each sample of submucosa was extracted using TRIzol reagent (Invitrogen, Thermo Fisher Scientific, Carlsbad, CA, USA). RNA quality was assessed using a spectrophotometer (Nanodrop 2000, Thermo Fisher Scientific, USA) at 260 nm absorbance and validated in 1 % agarose gel electrophoresis. cDNA was synthesized by reverse transcription polymerase chain reaction (**RT-PCR**) from mRNA using the Invitrogen Kit (Invitrogen, Carlsbad, CA, USA) following the manufacturer’s protocol.

### Real-time quantitative PCR (RT-qPCR)

Two µl of cDNA were mixed with 6 µl nuclease-free water, 10 μl of SYBR Green I Master (Applied Biosystems™ PowerUp™ SYBR™ Green Master Mix, Thermo Fisher Scientific, USA), and 1 μl of each forward and reverse primer. Forward and reverse primers, enzymes, and annealing temperatures for qPCR of the gene involved in oxidative phosphorylation are detailed in Table 1. The forward and reverse primers were designed using Primer3Plus (https://primer3.ut.ee/). RT-qPCR was performed using the LightCycler 480 Real-Time PCR System (Roche, Germany). Gene expression was quantified using the 2^−ΔΔCT method (Livak and Schmittgen, 2001), with expression levels normalized to *glyceraldehyde-3-phosphate dehydrogenase* (***GAPDH***).

**Table 1:** Primer sequences and their applications for qPCR.

Gene	5′ sequence 3′ forward/reverse primers	PCR product size (bp)	Annealing temperatures	Accession No.
*ATP5D*	5′– GCTGTCGCGATAGGCGCT–3′ 5′– TGTTTACCCGCGGCGTTTC–3′	102	58 °C	XM_040692427.1
*ATP5H*	5′–GCGCCAGAGGACGGTAGTGG–3′ 5′–CTCCTCGTTCCGCTCAGCTCC–3′	70	60 °C	XM_001232613.5
*ATP5J*	5′–GAAGCGCAGCGCCTACTTCC –3′ 5′–ATGACGGCACCGACGCTTTA–3′	162	62 °C	XM_040658784.2
*ACTN1*	5′–TGTTTACCCGCGGCGTTTC–3′ 5′–TCAGCCGCCTTCATTTCC–3′	60	60 °C	NM_204127.2
*SLC9A3R1*	5′–AGGGCCCGGACGGGTA –3′ 5′–CCACGTTCGTTCCGTCCACC–3′	147	56 °C	NM_001006424.2
*GAPDH*	5′–GGTGGCCATCAATGATCCCT–3′ 5′–CCGTTCTCAGCCTTGACAGT–3′	105	58 °C	NM_204305.1

Abbreviations: *ACTN1, actinin alpha 1; ATP5D, ATP synthase, H⁺ transporting, mitochondrial F1 complex, delta subunit; ATP5H, ATP synthase, H⁺ transporting, mitochondrial Fo complex subunit D; ATP5J, ATP synthase, H⁺ transporting, mitochondrial Fo complex subunit F6; SLC9A3R1, solute carrier family 9 member A3 regulator 1; GAPDH, glyceraldehyde-3-phosphate dehydrogenase*.

### SDS-PAGE and western blot analysis

SDS-PAGE was performed according to the procedure described by Chen et al. (2022) with minor modifications. Briefly, 0.5 g of jejunal submucosa was extracted and homogenized using RIPA Lysis Buffer (MedChemExpress, USA). After centrifugation, the concentration of proteins was assessed using a Microplate Spectrophotometer (Epoch, BioTek, VT) as described by the Lowry assay (Lowry et al., 1951). Samples were then loaded onto hand-cast 10 % acrylamide gels, and lane gels were cast with a stacking gel (4 % gel) and separating gel (10 % gel) to transfer protein to Nitrocellulose membrane (Whatman Protran; Dassel, Germany). After fractionation by SDS-PAGE, the membranes were incubated in a blocking buffer with 5 % skim milk prepared in Tris-buffered saline containing Tween 20 (TBST; 0.05 % Tween 20, 100 mM Tris-HCl, and 150 mM NaCl, pH 7.5). The membranes were then incubated with antibodies. The primary antibody used for detecting ACTN1 protein was Anti-ACTN1 antibody (1:5,000; RRID: AB_2227895; Catalog No. 11313-2-AP, Proteintech Group, Inc., Rosemont, IL, USA). The secondary antibody was HRP-conjugated Affinipure Goat Anti-Rabbit IgG (*H* + *L*) (1:5,000; RRID: AB_2722564, Catalog No. SA00001-2, Proteintech Group, Inc., Rosemont, IL, USA). Western blot data were imaged and analyzed using a ChemiDoc MP Imaging System (Bio-Rad Laboratories, Inc., Hercules, CA, USA). The relative protein expression was standardized with α-tubulin. Image analysis of Western blot bands was performed using ImageJ software (NIH, Bethesda, MD, USA).

### Morphology of tight junction in jejunum in transmission electron microscope technique

The jejunum tissues were cut approximately 1 × 1 mm and fixed with 1 % osmium tetroxide at 4 °C for 2 h. Following fixation, samples were dehydrated through a graded ethanol series: 70 %, 80 %, 90 %, and 95 % (each concentration applied twice), followed by absolute ethanol (100 %) three times. Each dehydration step lasted approximately 30 minutes. After dehydration, the tissues were infiltrated with propylene oxide twice and then embedded in resin. The resin embedded samples were polymerized by heating at 60°C for more than 24 h. The resin samples were sectioned at 50-70 nm in an ultramicrotome (Leica Ultracut R, Germany) and observed under a TEM. Tight junction length and the scale bar were measured using the integrated digital measurement tools within the TEM Server software (RADIUS, Version 2.1, Olympus Soft Imaging Solutions GmbH, Münster, Germany), with tight junction length expressed in nanometers (nm). Ultrastructural measurements of tight junction length were obtained from jejunal tissue samples (n = 3 chickens/group), with the average of three replicates calculated per sample.

### Statistical analysis

An independent samples t-test was performed to compare the means between LRFI and HRFI chickens for FE parameters, gene and protein expression levels, and tight junction length. All data are presented as mean values ± standard error of the mean (SEM), with statistical significance set at the 0.05 level (*P* < 0.05). Statistical analyses were performed using SPSS for Windows (IBM SPSS Statistics version 29.0, SPSS Inc., Chicago, IL, USA). Additionally, Principal Component Analysis (**PCA**) was performed using the Unscrambler X software (Camo Analytics, Oslo, Norway) to clarify and confirm the data, and to explore multivariate patterns and associations among molecular and phenotypic traits in chickens with divergent RFI. The input variables included gene expression levels of tight junction-related genes, ACTN1 protein expression, jejunal morphology parameters, as well as phenotypic traits such as BW, RFI, and FCR. All variables were mean-centered and scaled to unit variance prior to analysis. PCA biplots were used to visualize group separation and identify the major variables contributing to the observed variation.

## Results

### Growth performance and FE parameter

The growth performance and FE of KRC were compared between the two groups (Table 2). No significant differences were observed in body weight and MBW^0.75^ between the LRFI and HRFI chickens. However, RFI differed significantly (*P* < 0.001), with the HRFI group having a considerably higher RFI compared to the LRFI group. Furthermore, FCR was significantly higher (*P* < 0.001) in the HRFI group than in the LRFI group. These results indicate that the LRFI group exhibited superior growth performance and FE without any negative impact on body weight.

**Table 2:** Growth performance and FE parameters of different FE; HRFI and LRFI. Values are presented as mean ± SEM. Values with different superscripts are statistically different between treatment groups (*P* < 0.05).

Traits	HRFI group, n = 15	LRFI group, n = 15	P-value
BW, g	1450.466 ± 90.573	1392.333 ± 103.153	0.1244
MBW^0.75^, g	234.945 ± 11.506	227.816 ± 13.094	0.1245
FI, g	3501.800 ± 186.147	3032.300 ± 189.055	*P* < 0.001
RFI, g	230.017 ± 65.549	−212.907 ± 44.297	*P* < 0.001
FCR	2.508 ± 0.064	2.230 ± 0.065	*P* < 0.001

BW, body weight; FCR, feed conversion ratio; FE, feed efficiency; FI, feed intake; HRFI, high residual feed intake; LRFI, low residual feed intake; MBW⁰·⁷⁵, body weight to the power of 0.75; RFI, residual feed intake.

### Gene and protein expression in oxidative phosphorylation and tight junction pathways

**Fig. 1:**
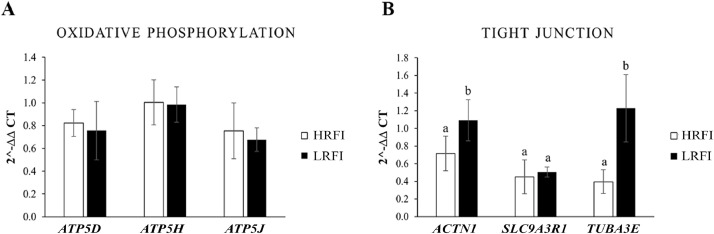
Effects of different FE of KRC on the expression levels of ATP5D, ATP5H, and ATP5J genes in the oxidative phosphorylation pathway (A) and ACTN1, SLC9A3R1, and TUBA3E genes in the tight junction pathway (B). Values with different superscripts are statistically different between treatment groups (*P* < 0.05). Abbreviations: ACTN1, actinin alpha 1; ATP5D, ATP synthase, H⁺ transporting, mitochondrial F1 complex, delta subunit; ATP5H, ATP synthase, H⁺ transporting, mitochondrial Fo complex subunit D; ATP5J, ATP synthase, H⁺ transporting, mitochondrial Fo complex subunit F6; FE, feed efficiency; HRFI, high residual feed intake; KRC, Korat chickens; LRFI, low residual feed intake; SLC9A3R1, solute carrier family 9 member A3 regulator 1; TUBA3E, tubulin alpha 3E.

Gene expression analysis revealed no significant differences (*P* > 0.05) in the mRNA expression levels of oxidative phosphorylation-related genes, including *ATP5D, ATP5H*, and *ATP5J*, between the LRFI and HRFI Chickens (Fig. 1A). These results suggest that mitochondrial energy production, as indicated by the expression of these genes, may not differ between the two groups. In contrast, significant differences were observed in the expression of tight junction-related genes. The LRFI group exhibited significantly higher (*P* < 0.05) mRNA expression levels of *ACTN1* and *TUBA3E* compared to the HRFI group, whereas no significant difference was observed in *SLC9A3R1* expression (Fig. 1B).

**Fig. 2:**
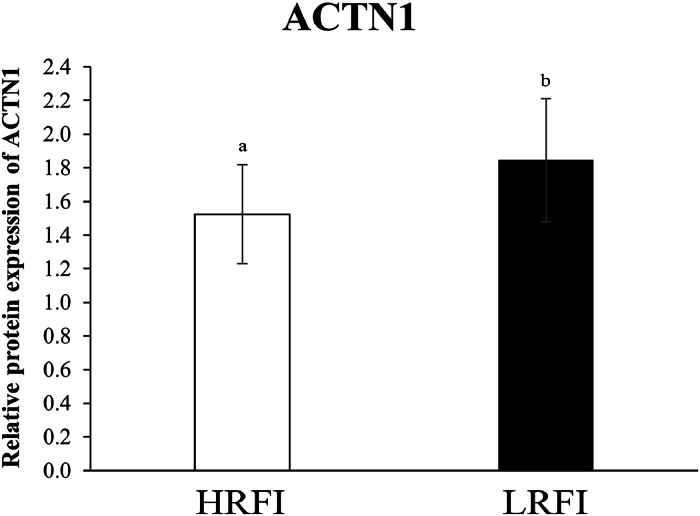
SDS-PAGE pattern of jejunal proteins extracted from Korat chickens with different FE levels. Western blot images showing ACTN1 protein expression of LRFI and HRFI chickens. Different letters indicate significant differences between groups (*P* < 0.05). Abbreviations: ACTN1, actinin alpha 1.

Western blot analysis showed that ACTN1 protein expression was significantly higher (*P* < 0.05) in the LRFI group compared with the HRFI group (Fig. 2). This result is consistent with the mRNA expression pattern, suggesting transcriptional regulation of *ACTN1* by FE status.

Together, these findings indicate that while oxidative phosphorylation-related gene expression does not differ significantly between FE groups, the upregulation of *ACTN1* and *TUBA3E* at the mRNA level and *ACTN1* at the protein level in LRFI chickens may be associated with enhanced tight junction structure and function, potentially contributing to improved nutrient absorption and FE.

### Morphology of jejunal tight junctions

**Fig. 3:**
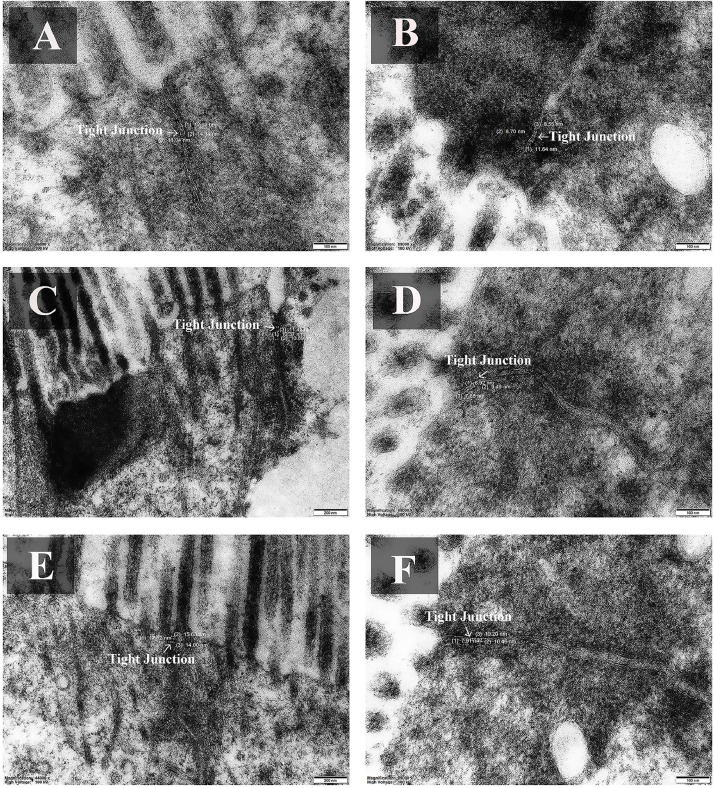
Effects of different FE on tight junction structures (TEM, x 89,000) in the jejunum between the HRFI group (A, C, and E) and the LRFI group (B, D, and F) of KRC. Representative tight junctions are indicated by arrows. Measurements of tight junction length (in nanometers) are shown where applicable. Scale bars: 200 nm for C and E; 100 nm for A, B, D, and F.

The morphology of tight junctions in the jejunum showed that the LRFI had greater tight junction length when compared with HRFI (Fig. 3). Interestingly, the average tight junction length was significantly shorter (*P* < 0.05) in the LRFI group (9.306**±0.473** nm) compared to the HRFI group (13.059**±2.367** nm).

### PCA of FE in relation to gene and protein expression and tight junction morphology

**Fig. 4:**
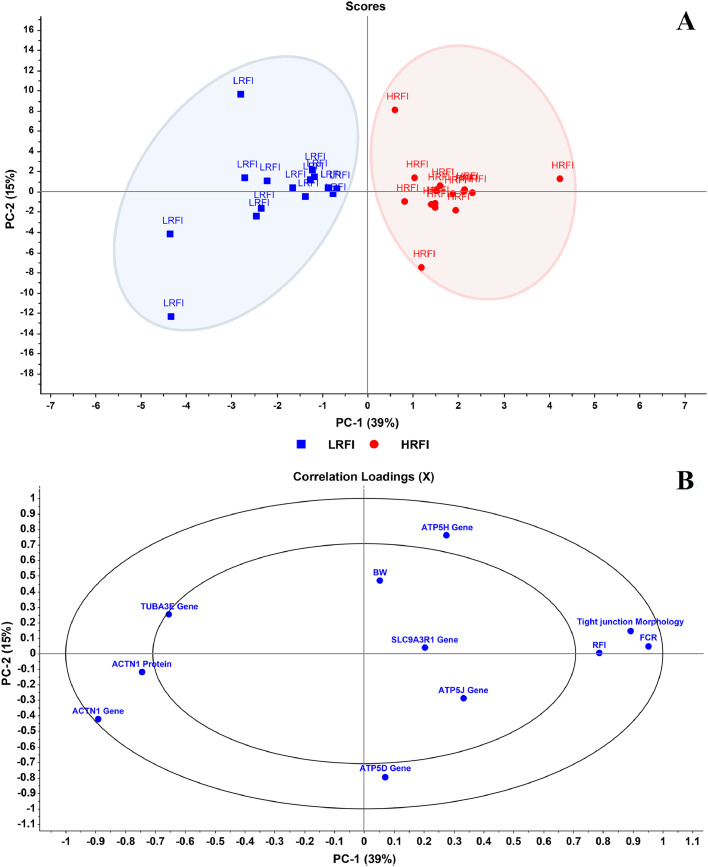
Principal component analysis (PCA) score plot (A) of PC1 versus PC2 for two experimental groups, and correlation loading plot (B) of PC1 versus PC2 showing gene expression, protein expression, and morphological variables, including tight junction morphology, body weight (BW), residual feed intake (RFI), and feed conversion ratio (FCR).

PCA was performed to assess the multivariate relationships among gene expression levels, tight junction-associated protein expression, intestinal morphology traits, and phenotypic performance indicators, including BW, RFI, and FCR in KRC. As chickens were classified into the LRFI and HRFI groups based on extreme RFI values, the PCA scores plot (Fig. 4A) showed a clear separation between the LRFI and HRFI groups along the first two principal components, PC1 and PC2, which accounted for 39 % and 15 % of the total variance, respectively. This result indicates distinct molecular and morphological characteristics associated with the two divergent FE phenotypes.

The PCA correlation loadings plot (Fig. 4B) revealed that genes and protein expression variables, particularly those related to tight junction integrity (e.g., *ACTN1* gene and protein, and *TUBA3E* gene), clustered on the negative side of PC1, whereas FCR, RFI, and tight junction morphology loaded positively. This inverse distribution indicates that LRFI chickens tend to have higher expression of tight junction-related genes and proteins, along with shorter tight junction distances. In contrast, HRFI chickens were associated with wider tight junctions and lower expression levels of these markers.

Collectively, these findings suggest that the expression of genes and proteins involved in tight junction architecture, along with jejunal morphological features, may contribute to differences in FE in slow-growing chickens. The separation of groups along PC1 underscores the potential of these molecular and structural indicators in identifying feed-efficient chickens in selective breeding programs.

## Discussion

This study provides novel insights into the molecular and structural mechanisms underlying FE in slow-growing chickens. For the first time, we demonstrate that variations in FE are associated with differential expression of genes and protein related to oxidative phosphorylation and tight junction pathways, along with distinct morphological differences in the jejunum. These findings highlight the importance of intestinal integrity in supporting improved FE in slow-growing chicken.

Both FCR and RFI values, as FE parameters between groups, showed significant differences between the LRFI and HRFI groups, with the LRFI group demonstrating superior FE. However, no significant differences in body weight or weight gain were observed between the two groups, indicating that the variation in FE was independent of growth performance. These findings are consistent with previous reports on FE in KRC (Poompramun et al., 2021; Sinpru et al., 2021; Kaewsatuan et al., 2022). Studies have noted that RFI has been used as a selection criterion to reduce FI and improve FE without affecting the body weight of slow-growing broilers and KRC (Wen et al., 2018; Sinpru et al., 2021). Therefore, RFI was considered a more suitable trait for selection in the present study.

To investigate the relationship between FE parameters and oxidative phosphorylation, the mRNA levels of *ATP5D, ATP5H*, and *ATP5J*, genes encoding structural subunits of mitochondrial ATP synthase (Complex V) essential for ATP production (Jonckheere et al., 2012), were analyzed in the jejunal tissue. No significant differences were observed between LRFI and HRFI chickens. The absence of transcriptional differences suggests that FE in KRC may not be primarily regulated at the transcript level in the jejunum. Given the jejunum’s primary role in nutrient absorption, its energy demands may remain relatively constant and be met through metabolic flexibility or substrate-level adaptation rather than transcriptional upregulation of ATP synthase genes (Kaewsatuan et al., 2022). Moreover, high-FE chickens have been reported to exhibit improved mitochondrial function and enhanced detoxification of reactive oxygen species (ROS), potentially reducing oxidative stress without requiring elevated transcription of oxidative phosphorylation genes (Schäff et al., 2012; Wang et al., 2019). These findings may support the development of a new hypothesis that mitochondrial efficiency in FE of chicken is regulated through mechanisms beyond gene expression, potentially involving metabolic pathways such as glycolysis, fatty acid β-oxidation, and the tricarboxylic acid cycle, which collectively contribute to more effective energy metabolism (Willems et al., 2013; Kong et al., 2016; Xu et al., 2016).

The functionality of tight junctions between epithelial cells is crucial for protecting physiological and biochemical barriers from external environmental factors and maintaining homeostasis (Panwar et al., 2021). In this study, the expression of *ACTN1* and *TUBA3E* genes was significantly higher in the LRFI group, while there was no significant difference in *SLC9A3R1* expression. *ACTN1* was selected for validation at the protein level because it showed the most pronounced differential expression at the transcript level among the tight junction-related genes investigated. Additionally, ACTN1 is a cytoskeletal protein that plays a critical role in anchoring transmembrane proteins of tight junctions to the actin cytoskeleton, which is essential for maintaining epithelial integrity and selective permeability (Noureddine et al., 2025). The elevated expression of ACTN1 protein is consistent with a previous proteomic study on KRC that reported upregulated expression of ACTN1, SLC9A3R1, and TUBA3E in high FE chickens (Kaewsatuan et al., 2022). Similarly, a transcriptome study found that genes encoding these proteins are upregulated in the LRFI epithelium of beef cattle (Kong et al., 2016). The high expression levels of *ACTN1* and *TUBA3E* in LRFI chickens suggest that these genes may be upregulated to protect, repair, and maintain the efficiency of tight junctions when there is an imbalance of biomolecules in the intestinal tract (Osselaere et al., 2013). Tight junctions have also been linked to variations in FE potential and improvements in carcass quality and immune response, contributing to a healthy gut (Groschwitz and Hogan, 2009; Zhang et al., 2023). Therefore, these findings suggest that the elevated expression of *ACTN1* and *TUBA3E* genes, along with an ACTN1-specific protein in LRFI chickens, may help maintain the efficiency and integrity of tight junctions in the jejunum, which could be a mechanism contributing to their superior FE.

The morphology of tight junctions in the jejunum revealed that LRFI chickens exhibited improved intestinal health, as evidenced by reduced tight junction length, compared to HRFI chickens. This was further supported by upregulation of associated genes, indicating that the difference in FE directly impacts the morphology of the tight junctions. Given that increased tight junction spacing is associated with a higher risk of pathogen translocation, which may compromise epithelial integrity and negatively affect FE (Hecht, 2001; Peng et al., 2017; Panwar et al., 2021). Thus, these findings suggest that the compact and stable tight junctions in LRFI chickens may contribute to better epithelial function and intestinal health, potentially supporting improved FE.

The PCA provided a comprehensive multivariate perspective on the relationship between gene and protein expression, jejunal morphology, and FE parameters in KRC. The clear separation along PC1 highlights major biological differences between LRFI and HRFI groups. In addition, tight junction-associated genes and protein, particularly *ACTN1* and *TUBA3E,* were important contributors to the variation in FE. The inverse loading directions of *ACTN1* and *TUBA3E* expression relative to RFI, FCR, and tight junction distance along PC1 suggest that higher expression of these markers is linked to better epithelial barrier function. The present findings support previous findings that high-FE chickens have reduced permeability and improved nutrient absorption (Panwar et al., 2021; Zhang et al., 2023).

Although genes associated with oxidative phosphorylation (*ATP5D, ATP5H,* and *ATP5J*) did not strongly load along the same axis as tight junction markers, their contribution to PC2 implies a more generalized role in cellular energy metabolism rather than a discriminating factor between FE groups. This is consistent with previous studies indicating that mitochondrial function may be regulated at post-transcriptional or metabolic levels, rather than solely through gene expression in the jejunum (Kong et al., 2016; Yang et al., 2020). Mitochondrial capacity for ATP synthesis and ROS detoxification has been strongly linked to improved FE, as demonstrated by studies highlighting mitochondrial bioenergetics as a critical factor in poultry growth and metabolic performance (Bottje et al., 2006). These findings suggest that mitochondrial energy regulation may still support overall metabolic efficiency, even in the absence of significant transcriptional shifts.

Notably, the close positioning of the *ACTN1* gene and protein expression in the loading plot supports the hypothesis of coordinated transcriptional and translational control, reinforcing its importance in cytoskeletal dynamics and tight junction stability. The inclusion of *TUBA3E,* a gene encoding a tubulin isoform involved in microtubule organization, further supports a structural adaptation in the intestinal epithelium that may enhance absorptive capacity and resilience in feed-efficient chickens.

Taken together, these PCA-based insights highlight the critical role of intestinal barrier integrity and cytoskeletal regulation in modulating FE in KRC. The integration of gene and protein expression data with intestinal morphology provides a multidimensional framework for identifying reliable biomarkers of FE. Such findings are valuable for refining genetic selection strategies in slow-growing poultry breeds, aimed at enhancing both productivity and gut health (Aggrey et al., 2010; Yi et al., 2018; Prakash et al., 2020).

## Conclusion

This study highlights the utility of RFI as a robust indicator for designing effective selection strategies. Our findings show that LRFI chickens exhibited an upregulation of tight junction-related genes (*ACTN1* and *TUBA3E*) and corresponding ACTN1 protein expression. This molecular evidence suggests that LRFI chickens possess stronger jejunal epithelial tight junctions, which may be a key factor contributing to their improved feed efficiency.

## Declaration of competing interest

The authors declare that they have no known competing financial interests or personal relationships that could have appeared to influence the work reported in this paper.

## Acknowledgments

The authors sincerely thank 10.13039/501100004352Suranaree University of Technology for providing financial support through the One Research One Graduate (OROG) program and this project is funded by 10.13039/501100004704National Research Council of Thailand (NRCT): NRCT5-RSA63009-05 through the Mid-Career Researcher Development Program. The authors would like to express their sincere gratitude to the Center of Excellence (CoE) on Technology and Innovation for Korat Chicken Business Development, Suranaree University of Technology Farm for their technical support and provision of experimental facilities. Special thanks are also extended to all research staff and students involved in animal care, sample collection, and laboratory analyses.

## Disclosures

The authors declare that they have no known competing financial interests or personal relationships that could have appeared to influence the work reported in this paper.

## CRediT authorship contribution statement

**Sukritta Kongthungmon:** Writing – review & editing, Writing – original draft, Visualization, Methodology, Conceptualization. **Saknarin Pengsanthia:** Writing – review & editing, Writing – original draft, Visualization, Methodology, Conceptualization. **Boonyarit Kamkrathok:** Writing – review & editing. **Pramin Kaewsatuan:** Writing – review & editing. **Wittawat Molee:** Writing – review & editing. **Amonrat Molee:** Writing – review & editing, Supervision, Resources, Project administration, Funding acquisition, Conceptualization.
